# The Prevalence and Socio-Demographic Correlates of Food Insecurity in Poland

**DOI:** 10.3390/ijerph17176221

**Published:** 2020-08-27

**Authors:** Hanna Dudek, Joanna Myszkowska-Ryciak

**Affiliations:** 1 Department of Econometrics and Statistics, Institute of Economics and Finance, Warsaw University of Life Sciences (WULS), 02-776 Warsaw, Poland; hanna_dudek@sggw.edu.pl; 2 Department of Dietetics, Institute of Human Nutrition Sciences, Warsaw University of Life Sciences (WULS), 02-776 Warsaw, Poland

**Keywords:** food insecurity, households, socio-demographic determinants, socioeconomic program

## Abstract

While food insecurity (FI) has been extensively studied in many countries, there have been few empirical contributions in Poland to date. The main objective of our research was to identify the socio-demographic factors affecting the risk of FI in Poland within 2014–2019. Moreover, we aimed to examine the effects of the family-oriented social program “Family 500+” by comparing the situation in various types of households with children a few years before and after the program was launched. The analysis was based on the set of eight-item FI indicators adopted by the Food and Agriculture Organization using the Gallup World Poll nationally representative survey data. Based on our results the most vulnerable groups in the context of FI were identified. We confirmed the importance of education, gender, age, marital status, household composition, status of employment and income in preventing FI. The effectiveness of the support program in reducing FI was demonstrated as households with at least three children experienced significant improvement in the FI status during the studied years. These findings should be especially important in the context of the impact of the COVID-19 pandemic on FI. As FI can affect the quality and quantity of food choices it is associated with a poorer health status, which increases the risk of infection, including COVID-19, and worsens recovery prognosis. Planning an efficient response to the pandemic requires a comprehension of the increased risk of exposure experienced by people, especially those who are food insecure.

## 1. Introduction

According to the World Health Organization (WHO) data, 821 million people in the world are undernourished. This means that one in nine of the world population habitually consumes an insufficient amount of food to meet individual dietary needs [1], thus experiencing food insecurity. According to the Food and Agriculture Organization of the United Nations (FAO), food insecurity (FI) is defined as a condition when people lack secure access to sufficient amounts of safe and nutritious food for normal growth, development and an active and healthy life [2]. In other terms, FI occurs when individuals and/or families in a household adjust their dietary intake or preferences because of a lack of physical or economic resources [3]. Although the basic needs of the human body are similar worldwide, the individual factors (e.g., age, gender, physical activity) determine an adequate supply of energy and nutrients [4]. Factors which may lead to FI include the non-availability of food, lack of access, improper utilization and instability over a certain time period [5]. On a household level, FI risk factors are primarily financial, but socio-demographic factors (education, race/ethnicity, family composition, etc.), time, employment skills, housing status, health status, food skills or capabilities, health insurance status, social support, past economic hardship, and the immediate food environment (availability of affordable nutritious food) may also influence the food security status [6]. FI can be analyzed in relation to its causes or effects, in qualitative or quantitative terms, whether the situation is real or potential and on a macro- or country-wide level, a meso-, micro- or household level [7]. It should also be noted that the impact of FI can be experienced not only by individuals but also by the health system and society [8].

However, FI is often analyzed at the household level due to the fact that food purchasing decisions are usually made at this level. FI is difficult to measure as it is a multifaceted problem, and households (and also individuals) can drift between food security and insecurity over time [9]. Several indicators and consequently methods for assessing FI have been proposed thus far to understand the problem and monitor progress in eliminating hunger, as well as to provide targets for national and international political action. However, so far there is no one generally accepted official measurement of food insecurity worldwide [9]. Although measuring FI based on individual data (people’s direct experience) are considered more reliable than model-based macro measurements [10]. 

### 1.1. Food Insecurity Measurement Methods

The FI status is often evaluated based on the module of questions (the Core Food Security Module, CFSM) developed by the United States Department of Agriculture (USDA) available on the USDA Economic Research Service website [11]. The full version of the questionnaire assesses the problem at a household level (18 questions) and contains specific questions (6) about the situation of children. There are also shorter versions (with 10 or 6 questions) and modified versions (for specific aspects of food insecurity) of the available USDA food insecurity module available [11,12]. Based on this module there are three ranges of FI severity on the household level: 1 = marginal, 2 = low and 3 = very low food security. Previously used categories of food insecurity included respectively: food insecurity without hunger, and food insecurity with hunger on a moderate and severe level. 

The other widely used and practical instrument for measuring FI proposed by the United Nations is the Food Insecurity Experience Scale (FIES) [13]. The FIES survey module contains 8 questions referring to the experiences of the individual respondent or of the respondent’s household as a whole. The questions focus on self-reported food-related behaviors and experiences associated with increasing difficulties in accessing food due to resource constraints. Developed by nutritionists, the FIES is based on a well-grounded construct of the experience of FI composed of three domains: worry/anxiety, changes in food quality and changes in food quantity [14]. Based on this module, the risk of FI can be identified in communities and individuals in a comparable way across different populations. The FIES measures the severity of food insecurity based on the number of positive answers to questions. The FIES score ranges from zero (no symptoms of FI) to 8 (all symptoms of FI). When analyzed in conjunction with the respondent and household characteristics, the FIES data can deepen the understanding of risk factors and consequences of FI for individuals and households [15]. Since 2014, the FAO has started monitoring food security by including the FIES in the Gallup World Poll (GWP), an annual survey conducted in over 140 countries worldwide. The FIES also serves as one of two indicators chosen to monitor Target 2.1 of the United Nations’ 2030 Agenda for Sustainable Development (Target 2.1 aims to end hunger and ensure access by all people, in particular the poor and those in vulnerable situations, including infants, to safe, nutritious and sufficient food all year round, by 2030) [16,17]. The FIES is a relatively new approach in measuring FI, but has been used numerous times for assessing FI in the global context [15,18,19] and in selected countries or regions [20,21,22].

### 1.2. Research Gap on Food Insecurity in Poland

Most research on FI in Europe is focused mainly on the situation in Western European countries, such as the United Kingdom [23], Ireland [24], and Germany [20]. This paper sheds a light on the situation of households in Poland, a central European middle-income country. Poland has successfully transformed from a centrally planned to a market economy, becoming a member of the European Union (EU) in 2004. During its membership in the EU, the Gross Domestic Product (GDP) per capita in Poland increased from $9610.4 in 2004 to $17386.9 in 2019, respectively. Compared to the remaining 10 countries that joined the EU in 2004, this gives Poland the 9th position, just behind Hungary and ahead of Latvia. Since joining the EU, the unemployment rate has dropped from 20% to 6%, which is slightly lower than the EU average. However, the median equivalised net income is still one of the lowest in the UE (monthly €548 versus €1473 in year 2018) [25,26]. 

While there is considerable research on the socioeconomic situation in Poland, especially on the various aspects of poverty [27,28], there is a shortage of studies in the field of FI. Therefore, it is important to look at the Polish population in terms of the inability to achieve the state of food security. This study aims to fill the gap in the existing literature regarding the prevalence and socio-demographic correlates of FI in Poland. Our results can not only be used for international comparisons, but also can be helpful in identifying groups at risk of FI in EU member states with a similar economic status for which information is not available. In addition, it aims to examine the effects of the social welfare program “Family 500+” launched by the Polish government in April 2016.

### 1.3. The “Family 500+” Program

The program was developed to increase the birth rate and reduce child poverty by improving the living conditions of large families. The birth rate in Poland is one of the lowest in the EU, and the reasons include economic factors: the problem of returning to work after giving birth, housing difficulties and insufficient support from the government [29]. On the other hand, in 2014, approximately 10% of children and adolescents under the age of 18 were in extreme poverty, and in households with four or more children this indicator reached almost 27% [30]. The program entitles parents to receive tax-free benefit of 500 PLN (around €120) per month for each second and subsequent child up to the age of 18, regardless of the household income level. In the case of a family income lower than 800 PLN net per person (or 1200 PLN net per person for families with a disabled child), this benefit may also be received for the first or only child in the family (more information on the “Family 500+” program and other family benefits can be found at [31,32]). The “Family 500+” child cash benefit was nearly three time higher than previous and still existing income tested family benefits [33]. At the time of introducing the program, it accounted for over 10% of the average gross monthly salary [34]. The recipients of benefits in the program are the parents, legal or actual guardians of the child. Thus, all types of families, including e.g., single parents, married couples, cohabiting parents, foster families, are allowed to apply for benefits. Almost four million children under 18 (55%) were covered by the program, with a higher coverage in rural than in urban areas. In particular, in 2017 the program covered 3.85 million children, as a result of which approx. 234,523 million PLN (around €5318 million) were transferred to families [35] and in the same year the rate of extreme child poverty fell from 9% in 2015 to 4.7% [36].

With the introduction of the “Family 500+” program, Poland has become one of the most social states in Central and Eastern Europe [37]. Before introducing the “500+” program Poland lacked any coherent family policy. Compared to other EU countries, Poland was characterized by a low total expenditure on family benefits and high poverty among households with children. According to Eurostat data [38], in 2015, before the program was introduced, the overall expenditure on social protection for families and children in Poland was – as a percentage of the GDP – one of the lowest in the EU, whereas in 2017 it was slightly above the EU average. What is more, in 2017 the share of social protection expenditure on family benefits in Poland was one of the highest in the EU. 

However, the “Family 500+” is criticized by some economic and political milieus because of its low effectiveness in improving the birth rate and the long-term deterioration of the state’s public finances. Some experts point to other, less costly, instruments supporting families with children, e.g., support for pre-school education and care, encouragement of fathers’ involvement in child-raising, flexible employment for young parents, etc. [39]. They postulate developing social policy and creating a systemic support for families and citizens of all ages instead of distributing state money only for a certain group of Poles. Nevertheless, the “Family 500+” program is an example to other countries how to boost economic stability among families. In 2018, Lithuania introduced the universal child benefit, so-called “child money” in the amount of €30 paid to all children from birth until the age of 18 [40]. Undoubtedly, such support programs for children are a social shield that will sustain the family’s income, therefore it is worth analyzing their effectiveness. 

Despite the fact that in the literature there are studies on the impact of the “Family 500+” on poverty, material deprivation and the labor market [39,41,42,43,44], the results of analyses regarding the efficiency of this program in terms of FI have not been presented so far. This issue is particularly important in terms of child malnutrition and the lasting consequences of experiencing food insecurity during childhood. Therefore, our study addresses this issue. The results obtained in this regard may contribute to shaping future social protection for families and children in Poland and be an inspiration to policy makers in other countries.

To our best knowledge, this study significantly contributes to the literature on food insecurity by providing the first evidence on the prevalence and correlation of FI in Poland based on the FIES data collected by the FAO using the Gallup World Poll. In addition, it sheds light on the changes in the FI status within years 2014–2019 in the entire Polish population and in various socio-demographic groups, with particular emphasis on individuals from households with children.

## 2. Materials and Methods 

The 2014–2019 GWP data encompassing yearly about 1000 individuals aged at least 15 was used in the study. More specifically, in 2014–2018 the sample size was 1000, and 1080 in 2019. The survey questions were asked to a nationally representative sample during face-to-face interviews. The sample was drawn proportional to the population, and the country was stratified by region and by population size strata [30,45,46]. 

The individual-level food insecurity status was measured using the FIES, an experience-based measure of FI developed by the FAO Voices of the Hungry project [13] and incorporated into the annual GWP for the first time in 2014 (the last available data is for 2019). The FIES Survey Module is composed of eight questions with dichotomous yes/no responses. These questions relate to the individual’s experience with FI during the previous 12 months. Table 1 lists the eight questions of the FIES module.

Responses are coded as 1 for “yes”, 0 for “no” and “N/A” otherwise, wherein the percentages of N/A are very low. Thus, in our study responses “yes” recoded as 1, all other responses are recorded as zero. The FIES raw score was calculated by summing the number of affirmative responses. Food security status is based on the individual’s raw score. 

The data was analyzed in two-year periods: (1) 2014–2015, (2) 2016–2017, and (3) 2018–2019. This approach allowed to assess the impact of the “Family 500+” social program on FI by comparing the situation in various types of households with children. The first period covered the time before the program was introduced, the second was a transition period, while the third covered the period when the socioeconomic reforms were consolidated. Additionally, such an approach reduced the impact of the year-to-year sampling variability [3].

### 2.1. Dependent Variable

In our study we focused on the dichotomous variable: being food secure versus being food insecure. More precisely, FI was divided into two categories: (1) “Food Secure” (the FIES raw score of zero) and (2) “Food Insecure” (the FIES raw score of one or more). In all analyses, the food insecure status was used as the reference group.

### 2.2. Correlates

The set of correlates consisted of socio-demographic characteristics including educational level, gender, age, marital status, location of dwelling, and household composition. These are typical variables available in the GWP database and used in FI studies worldwide [15,18,19]. 

The educational level was classified as elementary or lower (elementary), secondary (secondary), and high or higher (tertiary). Marital status was classified as never married, married, living with partner, divorced or separated, or widowed. The place of residence was categorized as either city, town, rural area, or farm based on the participant’s current address. Household composition included one-person households, households of two adults, households of at least three adults, households with one child, households with two children, households with at least three children. 

In addition, economic variables such as the income quintile group and employment status were included. The employment status was classified as full time employed for an employer, full time self-employed, out of workplace, part-time employee and unemployed. 

### 2.3. Data Analysis

Statistical analyses were conducted using the STATA 14 statistical software (StataCorp LP, College Station, TX, USA). Sampling weights provided by the GWP were used to adjust for the sampling effect in all statistical analyses, and all presented results were weighted estimates. An initial descriptive analysis was performed for every independent variable, determining frequencies and averages in the two groups of respondents: food secure and food insecure. The associations between FI and categorical (nominal) socio-demographic variables were assessed by calculating the chi-square for each variable as a whole. Cramer’s V measure was used to assess the strength of this association. Differences in the continuous variables between corresponding groups (FI = 0 and FI = 1) were determined using the t-test, and additionally the size index of Cohen’s d effect was calculated. Subsequently, multiple logistic regression analyses were carried out to explore differences in FI according to the demographic, social and economic characteristics of respondents. Regression models should not include overly correlated variables together, which could distort the results [47,48]. Following this recommendation, we applied a rule of thumb calling association weak for values of association below 0.25 [49,50]. Therefore, independent variables were included in the models, for which the pairwise Cramer’s V measure did not exceed 0.25 in all analyzed periods. In other words, values at least equal to 0.25 were treated as indicating moderate to strong association, while values less than 0.25 as indicating weak association. For all tests, *p* < 0.05 was considered as significant.

## 3. Results

**Figure 1:**
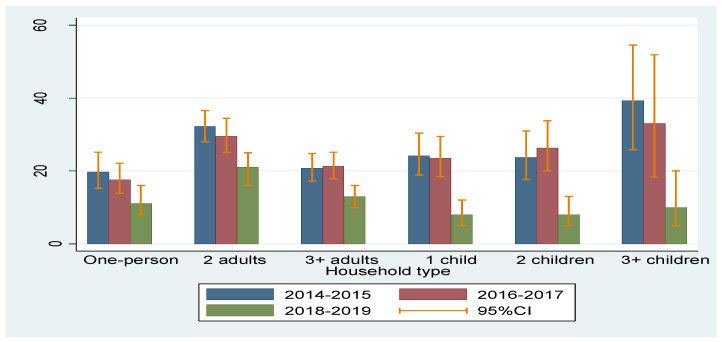
The prevalence of FI according to the household composition.

The prevalence of any FI in Poland ranged between 23.9% in 2014–2015 and 12.1% in 2018–2019. The prevalence of FI among individuals living in households of various compositions is presented in Figure 1.

The situation of respondents living in households with at least three children has improved the most. In such households, the prevalence of FI decreased from approx. 39% in 2014–2015 to 10% in 2018–2019, which means a nearly fourfold decrease. In households with one and two children, a three-fold decrease in the prevalence of FI was recorded, while in households without children it decreased by less than twofold.

Table 2 presents the social, demographic and economic characteristics of the two studied groups depending on their FI status.

An uneven distribution of the majority of characteristics was observed depending on the FI status. Especially respondents with tertiary education less often showed FI compared to individuals with secondary education, while those with elementary education relatively more often. As might be expected, the FI status was tightly linked to income. In the higher income quintile group, FI was less common. However, it should be underlined that no perfect relationship was observed. FI also affected people who are not living in poverty. Even among 40% of the richest over 20% of respondents experienced FI. On the other hand, among 40% of the poorest over 30% of did not report any FI. Statistical analysis provided more detailed information on the association between FI and respondents characteristics. Subsequently, bivariate analyses were performed, including the chi-squared test and the Student’s t-test for independent samples, depending on the type of variable (Table 3).

Significant differences (*p* < 0.05) were observed between FI and the majority of the examined variables, namely: education, gender, age, marital status, household composition, status of employment and income. However, for location of dwelling no significant differences were observed in any of the analyzed periods. 

Cramer’s V scores suggested that among categorical variables education and income showed the strongest association with FI. In contrast, the weakest relationship was recorded to gender and place of residence. In the next stage of the study, logistic regression was used to assess differences in individual characteristics and explore correlations between FI and respondents’ characteristics. However, due to the moderately strong association between household composition and marital status, and between education and employment status we estimated models that did not contain highly correlated variables (the detailed information on pairwise correlations values between various respondents characteristics are presented in Appendix A: Table A1, Table A2, and Table A3). Thus, we decided to present two models; each of these models includes independent variables characterized by a weak pairwise association. In both model’s estimation was made controlling income quintiles.

Table 4 and Table 5 present the logistic regression results for further understanding. As the purpose of the study was to examine the effects of the “Family 500+” program, Table 4 presents the results of the logistics model estimation for a set of variables containing household composition, age and education.

The results presented in Table 4, show that there was a significant improvement (compared to two-adult households) in the FI status in households with children. For the 2018–2019 period, the odds ratios of experiencing FI were statistically significantly lower for households with children compared to two-adults households. It was observed that households with at least three children significantly improved their position in relation to two-adult households. Moreover, it was found that:

- there was an evidence that women were more likely to be food-insecure than men;
- elementary and secondary education were associated with an increased odds ratio of FI compared to tertiary;
- as income increased, the odds ratios declined;
- ORs less than 1 for squared term of age implied an inverted U-shaped effect. In other words, respondents reported being less FI when they were younger and older than when they were middle-aged.

All results are interpreted in a ceteris paribus way, meaning no other variable changes in order to obtain only the influence of the particular variable. In addition, Table 5 presents the results of logistic regression estimation containing marital status instead of household composition, and employment status instead of education.

The results in Table 5 show that divorced, separated, or widowed respondents presented a higher ORs compared to married respondents. On the contrary, being single was related to a lower ORs. Taking into account employment status, it was found that in 2014–2015 and 2016–2017 periods, the unemployed exhibited a significantly higher risk of being food-insecure compared to other groups. However, this regularity was not confirmed in the 2018–2019 period. 

## 4. Discussion

The obtained results regarding the association of socio-demographic factors with FI are consistent with other studies to a large degree. Our findings confirm the importance of such attributes as: gender, age, educational attainment, economic activity, marital status and income situation. As in other research [18,22,51], a higher prevalence of FI among women compared to men was found. Our results also confirm the inverted U-shape relationship between the age of the respondent and the prevalence of FI reported for upper middle-income and high-income countries [15,19,52]. In line with previous studies [19,22,53], our results confirm the role of education as an extremely important factor against FI. Moreover, it was found that usually unemployed individuals were the most vulnerable to FI. However, this regularity was not confirmed in 2018–2019. This result may be related to a small number of unemployed people in Poland during this period. According to Eurostat data [26], the unemployment rate systematically decreased in the analyzed period, not exceeding 4% within 2018–2019. As in Grimaccia and Naccarato [15], and Smith and Rabbitt [19], we found that separate, divorced or widowed respondents presented a higher probability of experiencing events of FI comparing to married respondents. However, unlike the above research, our results indicate that singles were not more affected by FI than married respondents. This is in line with results of Grimaccia and Naccarato [22], showing that in Eastern Europe the difference between these two groups was not significant at 0.05. Considering the observed inverted U-shape relationship between the age of the respondents and FI, our results can be largely explained by the age of respondents, as the average age in the never married group was 27, while in the married respondents group it was 50. Unlike in Grimaccia and Naccarato [15], no significant rural-urban differences were found in our study. Such differentiation exists in less developed countries [19]. However, in the upper-middle income and high-income countries, the FI difference between rural areas and towns and cities is often not statistically significant at 0.05 [19,22]. 

Our results show an association between FI and household composition, with particular emphasis on children. In 2014, children of large families were ten times more exposed to extreme poverty (18.1% versus 1.8%) compared to children from families with one child. In 3% of households with children, the financial conditions did not allow to provide a daily portion of vegetables and fruit, and in 2.9% a meal with meat (or its equivalent) [54]. It should be stressed that extreme poverty can lead to biological wasting and irreversible consequences in the functioning of the individual [55]. In poverty-stricken families, the diet is usually poor and unbalanced, children do not get breakfast at school, and cannot afford to buy lunch. Malnutrition, in addition to its health effects, leads to worse results in school, as research shows that it affects child concentration and prevents effective learning [56]. On the other hand, poverty is strongly related to overweigh and obesity. Findings from the UK Millennium Cohort Study [57] show that adolescents in the lowest income quintile were 2.1 and 4.1 times more likely to be overweight and obese compared to adolescents in the highest income quintile. Our study provides evidence that adults with at least three children experienced meaningful improvement during the studied years. The prevalence of FI in this group declined nearly fourfold in 2018–2019 in comparison to 2014–2015. This is probably due to the introduction of the “Family 500+” program in 2016, supporting mainly large families. According to Liszatyński [58] the cash benefit is mainly spent on meeting basic needs, such as food and clothing, as well as education and activities for children. Preliminary data analysis of household budgets in 2016 [42] showed that the benefit not only reduced poverty, but it also improved the consumption structure of all members of households with children. These patterns of spending the benefit confirm its investment character, i.e., improving the health status and education of children, which translates into an improvement in the quality of life of future generations. There is strong evidence that cash transfers are likely to improve food security in respect of food consumption and diet quality [59,60]. However, their impact on nutrition and nutritional status of individuals (especially children) is rather inconclusive and needs more research [60]. 

Generally, for the whole population a twofold reduction in the prevalence of FI in 2018-2019 in comparison to 2014–2015 was observed. This may be related to the generally improving situation in the country in 2014–2019. Poland’s economy maintained rather strong and stable growth in 2014–2019. Poles experienced an boost in revenues, employment, and wages [26]. However, the decrease of FI was not proportionate in all groups. In particular, a relatively greater improvement was recorded among people living in households with children than among people living in households with adults. Thus, our study revealed the importance of social child support in reducing FI.

According to Alkire et al. [61] deprivation in nutrition predicts a high risk from COVID-19 and there is a growing body of evidence that FI is associated with a poorer diet quality [62]. Drewnowski et al. [63] showed that the cost of the food decreases with the increasing share of fats and sugar. Cheap highly processed energy-dense food products are high in saturated fats and added sugars, with a low content of essential and often deficient in the diet nutrients: vitamins, minerals and phytochemicals. Such a diet can cover the body’s energy requirements, but at the same time results in a reduced intake of micronutrients and deficiencies of selected vitamins or minerals (e.g., vitamin A, D, calcium, iron, iodine, zinc, etc.). Shifting towards more starchy foods and away from micronutrient rich vegetables, fruits, meat, and dairy products can lead to various forms of malnutrition with negative long-term effects [62,63,64]. An impaired nutritional status can affect the body’s defence functions and increase susceptibility to infections [65]. This can affect all populations groups, but children and pregnant women are particularly at risk. For example, children and pregnant women from insecure households had almost 3.0 times higher odds of experiencing iron-deficiency anemia [55], which among other health consequences, damages immune mechanisms [66]. On the other hand, the pandemic lockdown negatively affected the global and domestic economy, which may increase the incidence of FI, especially in risk groups. The COVID-19 pandemic has also put strain on food supply chains, with bottlenecks in farm labour, processing, transport and logistics, as well as momentous shifts in demand. However, not all sectors and food products have been equally affected, and different products have experienced disruptions at different stages of the supply chain. Transport and logistics problems was most pronounced for perishable high nutrients-value products, such as fruits and vegetables whereas cereals were not affected [67]. Thus, at the country level, there is a need to monitor domestic food and agricultural supply chains, track how the loss of employment and income is impacting people’s ability to buy food and ensure that food systems continue to function despite COVID-19 challenges. Government should provide special procedures for food, trade and agricultural inputs to ensure supply chains are kept open and functional. 

Therefore, a stable cash benefit can provide a sense of security, especially in the poorer groups. Future research is highly advisable to explore and compare the FI status in Poland with FI in other EU countries, in particular from Central and Eastern Europe.

### 4.1. Policy Recommendations

The results of our study provide guidelines for prioritizing policy and appropriate responses to FI. Evidence based on the nationally representative sample is essential to analyze the problem of FI. Our findings enable the identification of socially vulnerable groups with respect to their food and nutrition situation. Knowledge about individual factors influencing FI is crucial to formulate responsible and effective policies dedicated to relieving individual food insecurity. Policy and intervention planning should be more targeted at those in need. 

Our results suggest that the government’s “Family 500+” social program has provided meaningful help for people living in households with at least three children. Thus, it is important to ensure the long-term continuation of this program. Especially now, during the pandemic crisis, it will be useful as a powerful social shield that will sustain the income of households and help those most in need, i.e., large families. However, there is a strong need for more data and evidence to establish the detailed impact of the “Family 500+” program on FI. Our study also reveals that poorly educated adults living in one-person households faced higher FI compared to the national average. Despite the improvement of their situation within the 2014–2019 period, these individuals were, on average, disproportionately affected by FI. Therefore, policies should be targeted not only at households with children but also some groups of one-person households, especially widowed, poorly educated with low income. Such people need to be supported not only by government programs but also by various non-governmental organizations (social welfare associations and foundations, e.g., Caritas Polska, Polska Akcja Humanitarna Pajacyk, Kulczyk Foundation). So far, in Poland there is no coordinated policy in this respect. Cooperation of government agencies and non-profit organizations, such as food banks, could decrease the prevalence of FI (The Federation of Polish Food Banks consists of 31 Food Banks and distribute an average of 80,000 tons of food annually to over 1.6 million people in need, through 3500 aid organizations and community institutions. More information can be found at [68]). Thus, a new policy platform is needed where the challenges regarding FI could be identified. Such a platform would enable the creation of targets and goals for reaching policy objectives. New operational approaches should be experimented to address food security in Poland. A strong focus of joint interventions should be undertaken diversifying and adapting social protection strategies.

As the concrete steps have to be taken to decrease FI in Poland, below there are some recommendations should be considered by policy makers:

- An individual-level food security should get much greater policy consideration.
- The state of FI should be systematic monitored by state agencies and researchers.
- The assessment of FI must be backed up by evidence based on high-quality research and expert judgment.
- The social policy and a systemic support for all citizens at risk of FI should be provided. Especially, by considering the long-term continuation of the “Family 500+” program during COVID crisis as a powerful social shield that can sustain household’s income and help the most needy, e.g., large families.
- Cooperation of government agencies and various non-governmental organizations to reduce FI should be developed.

### 4.2. Strengths and Limitations 

This study adds to the understanding of FI and its correlates in Poland. This is the first study, to our knowledge, exploring FI in Poland on the basis of GWP data. The strengths of GWP data include the use of validated measures consistently across almost all over the world. GWP samples are nationally representative of the resident, non-institutionalized population aged 15 years and older in each country. Since 2014, they include FIES data which has been accepted worldwide as a valid and reliable measure of FI. One of the important advantages of the data used in the study is that it takes into account diet quality and reduced food quantity. Moreover, the FIES captures psychosocial elements associated with anxiety or uncertainty regarding the ability to procure enough food [13]. Furthermore, individual level data analysis gives the opportunity to study the characteristics of population groups at a greater risk of FI. Thus, it offers important policy instruments. This is particularly important in the context of the COVID-19 pandemic [69]. 

Despite these strengths, several limitations should be mentioned. First, GWP data classifies people under 15 as children, while children aged 15–18 are treated as adults. This limits the full assessment of the “Family 500+” program’s impact, as this program provides support to individuals up to and including 18 years. In addition, despite the fact that the analyzed sample of data is representative of the entire country, in some socioeconomic cross-sections it may not be sufficient. In particular, this issue concerns household composition. Therefore, to investigate the effect of the “Family 500+” program on FI, more detailed research should be performed. Secondly, this study relied on characteristics included in the GWP. However, it does not include factors which might be interesting, but they are missing from the GWP database. In particular, it would be worth considering in the further research the biological type of households, i.e., whether the household consists of parents and children, or if it is a multi-generational unit, etc. In addition, the GWP lacks detailed information on the financial situation of respondents, e.g., whether they have savings or debts, which can also affect FI. The cross-sectional nature of the study allows to identify associations. However, no causal inferences can be made and no changes over time can be identified. Therefore, it is not known whether individuals are permanently subject to FI or whether they experience incidental FI in a given year. 

## 5. Conclusions

This study is one of only a few studies examining FI in European countries. The study provides original evidence concerning the prevalence FI and its correlates in Poland, using the FAO Food Insecurity Experience Scale data. It has revealed that incidents of FI in Poland decreased in 2018–2019 compared to 2014–2015. The decline of FI could have been associated with an improvement in the general living conditions of the population in Poland during this period. A relative improvement in the status of people with at least 3 children was found compared to people in households consisting of two adults. This change may be related to the introduction of the “Family 500+” program in 2016. It can therefore be concluded that the program objective regarding the improvement of conditions of large families has been achieved. To combat FI and its associated consequences, an understanding of the factors influencing this phenomenon is required. This study revealed the relationship between FI and various socio-demographic and economic factors. In particular, FI was associated with a lower level of education, lower income, being a woman, divorced, separated, or widowed, unemployed and living in a one-person household. These population groups should be especially taken into account when designing social programs due to the greater risk of FI. Such support programs may significantly decrease the FI of large families, which in turn may have a positive impact on the growth and development of children, especially those from families at risk of poverty. 

## Figures and Tables

**Table 1:** Questions in the food insecurity experience scale (the FIES).

During the Last 12 Months, Was There a Time When, Because of Lack of Money or Other Resources:
1. You were worried you would not have enough food to eat?
2. You were unable to eat healthy and nutritious food?
3. You ate only a few kinds of foods?
4. You had to skip a meal?
5. You ate less than you thought you should?
6. Your household ran out of food?
7. You were hungry but did not eat?
8. You went without eating for a whole day?

**Table 2:** The prevalence of food insecurity according to respondent’s characteristics (food secure FI = 0, food insecure FI = 1). Standard errors for the proportions are presented in parentheses.

Variables	2014–2015		2016–2017		2018–2019	
	FI = 0	FI = 1	FI = 0	FI = 1	FI = 0	FI = 1
Total group	*n* = 1506 (76.1%)	*n* = 494 (23.9%)	*n* = 1567 (77.6%)	*n* = 433 (22.4%)	*n* = 1818 (87.9%)	*n* = 262 (12.1%)
Household composition						
One-person household	14.3% (0.8%)	21.5% (1.7%)	10.0% (0.6%)	14.6% (1.5%)	11.9% (0.7%)	22.4% (2.6%)
Two adults	29.7% (1.3%)	24.8% (2.3%)	29.5% (1.2%)	27.7% (2.4%)	34.5% (1.2%)	36.2% (3.4%)
Three or more adults	25.8% (1.5%)	20.2% (2.5%)	30.7% (1.4%)	22.7% (2.6%)	26.4% (1.3%)	24.7% (3.6%)
H. with one child	16.3% (1.2%)	16.6% (2.1%)	16.9% (1.1%)	18.0% (2.2%)	14.8% (0.9%)	8.7% (1.9%)
H. with two children	10.9% (1.0%)	10.8% (1.6%)	11.1% (0.9%)	13.8% (2.0%)	10.0% (0.8%)	6.2% (1.6%)
H. with at least three children	3.0% (0.5%)	6.1% (1.4%)	1.8% (0.4%)	3.1% (1.0%)	2.4% (0.4%)	1.8% (0.7%)
Gender						
Male	49.6% (1.5%)	42.4% (2.7%)	49.1% (1.4%)	43.0% (2.8%)	49.2% (1.3%)	38.0% (3.5%)
Female	50.4% (1.5%)	57.6% (2.7%)	50.9% (1.4%)	57.0% (2.8%)	50.8% (1.3%)	62.0% (3.5%)
Education						
Elementary	13.5% (1.2%)	29.4% (2.6%)	13.1% (1.1%)	24.6% (2.7%)	13.8% (1.2%)	28.6% (3.7%)
Secondary	64.6% (1.5%)	64.5% (2.6%)	65.4% (1.4%)	66.7% (2.7%)	65.7% (1.3%)	64.9% (3.7%)
Tertiary	21.9% (1.2%)	6.1% (1.1%)	21.5% (1.1%)	8.7% (1.2%)	20.5% (1.0%)	6.5% (1.6%)
Marital status						
Married	56.2% (1.5%)	50.2% (2.7%)	58.6% (1.4%)	55.7% (2.7%)	61.7% (1.3%)	52.4% (3.6%)
Widowed	8.6% (0.8%)	13.2% (1.5%)	8.4% (0.7%)	14.1% (1.7%)	7.2% (0.6%)	20.6% (2.8%)
Single (never been married)	29.1% (1.5%)	22.4% (2.4%)	26.9% (1.3%)	17.9% (2.1%)	22.5% (1.1%)	13.4% (2.5%)
Divorced or separated	5.2% (0.6%)	13.6% (1.6%)	3.4% (0.4%)	7.8% (1.5%)	5.2% (0.5%)	7.1% (1.4%)
Domestic partner	0.9% (0.2%)	0.6% (0.4%)	2.6% (0.5%)	4.4% (1.1%)	3.4% (0.4%)	6.5% (1.6%)
Employment						
Unemployed	3.2% (0.5%)	10.9% (1.6%)	1.2% (0.3%)	8.0% (1.6%)	1.7% (0.4%)	4.4% (1.7%)
Part-time employee	7.4% (0.8%)	7.0% (1.4%)	4.5% (0.6%)	6.2% (1.5%)	4.3% (0.5%)	5.6% (1.7%)
Out of workforce	39.2% (1.5%)	50.3% (2.7%)	38.8% (1.4%)	46.0% (2.8%)	40.4% (1.3%)	52.5% (3.6%)
Full time self-employed	6.4% (0.7%)	3.8% (1.2%)	5.5% (0.7%)	4.3% (1.3%)	3.3% (0.4%)	1.0% (0.8%)
Employed full time for an employer	43.8% (1.5%)	28.0% (2.5%)	50.1% (1.4%)	35.5% (2.6%)	50.3% (1.3%)	36.5% (3.4%)
Income quintile group						
First quintile group	14.9% (1.2%)	35.4% (2.6%)	15.1% (1.1%)	37.0% (2.8%)	17.8% (1.1%)	35.8% (3.6%)
Second quintile group	18.4% (1.2%)	25.5% (2.3%)	20.2% (1.2%)	19.3% (2.1%)	19.8% (1.1%)	21.4% (2.9%)
Third quintile group	20.9% (1.3%)	17.2% (2.1%)	20.7% (1.2%)	17.4% (2.0%)	20.4% (1.1%)	17.2% (2.6%)
Fourth quintile group	21.5% (1.2%)	15.5% (1.8%)	20.5% (1.1%)	18.2% (2.1%)	20.4% (1.0%)	17.2% (2.8%)
Fifth quintile group	24.3% (1.2%)	6.4% (1.1%)	23.5% (1.1%)	8.0% (1.4%)	21.7% (1.0%)	8.5% (1.6%)
Location of dwelling						
City	28.2% (1.4%)	31.4% (2.5%)	31.4% (1.3%)	25.9% (2.3%)	33.4% (1.2%)	31.6% (3.3%)
Town	51.8% (1.5%)	50.8% (2.7%)	47.3% (1.4%)	53.6% (2.8%)	50.0% (1.3%)	53.8% (3.6%)
Rural area or a farm	20.0% (1.3%)	17.7% (2.0%)	21.3% (1.2%)	20.5% (2.3%)	16.6% (1.0%)	14.6% (2.4%)

**Table 3:** Association between food insecurity and respondents characteristics.

Variables	2014–2015		2016–2017		2018–2019	
	χ^2^ Statistics	Cramer’s V	χ^2^ Statistics	Cramer’s V	χ^2^ Statistics	Cramer’s V
Education	99.8643 *	0.2235	54.7420 *	0.1654	213.6431 *	0.1875
Gender	8.7498 *	0.0661	3.7105 *	0.0431	8.5438 *	0.0729
Marital status	71.3536 *	0.1889	35.8146 *	0.1338	67.7044 *	0.1804
Household composition	31.5470 *	0.1256	20.4791 *	0.1012	26.4947 *	0.1129
Location of dwelling	4.1054	0.0260	5.7812	0.0538	0.3467	0.0129
Status of employment	87.2894 *	0.2089	71.6900 *	0.1893	29.3828 *	0.1189
Income quintile	139.0146 *	0.2636	129.0974 *	0.2541	62.6946 *	0.1736
Age	t = 2.9388 *	Cohen’s d = 0.1527	t = 4.2891 *	Cohen’s d = 0.2335	t = 3.3725 *	Cohen’s d = 0. 2715

* means statistical significance at 0.05.

**Table 4:** Logistic regression models on food insecurity by set of variables including household composition, age and education.

Variables	2014–2015		2016–2017		2018–2019	
	OR	SE	OR	SE	OR	SE
Household composition (reference: two-adults household)						
One-person	2.457 *	0.402	1.823 *	0.318	1.740 *	0.364
Three or more adults	0.759	0.160	0.646	0.145	0.562 *	0.153
H. with one child	0.888	0.206	0.865	0.202	0.351 *	0.109
H. with two children	0.610	0.158	0.745	0.197	0.203 *	0.077
H. with at least three children	0.824	0.318	0.803	0.366	0.193 *	0.097
Gender (reference: male)						
Female	1.323 *	0.182	1.371 *	0.189	1.660 *	0.277
Age	1.091 *	0.022	1.107 *	0.025	1.071 *	0.029
Age squared	0.999 *	0.0002	0.999 *	0.0002	0.999 *	0.0003
Education (reference: tertiary)						
Secondary	2.768 *	0.599	2.069 *	0.376	2.616 *	0.730
Elementary	5.260 *	1.471	3.599 *	0.994	3.927 *	1.486
Income quintile group (reference: first quintile group)						
Second quintile group	0.586 *	0.115	0.360 *	0.071	0.420 *	0.099
Third quintile group	0.324 *	0.070	0.357 *	0.072	0.319 *	0.081
Fourth quintile group	0.284 *	0.064	0.343 *	0.074	0.266 *	0.076
Fifth quintile group	0.099 *	0.027	0.114 *	0.032	0.111 *	0.033
Constant	0.041 *	0.022	0.039 *	0.023	0.049 *	0.036

OR–Odds Ratio, SE–robust standard errors (with heteroscedasticity-robust asymptotic variance), * means statistical significance at 0.05.

**Table 5:** Logistic regression models on food insecurity by set of variables including marital status and employment status.

Variables	2014–2015		2016–2017		2018–2019	
	OR	SE	OR	SE	OR	SE
Marital status (reference: married)						
Widowed	1.643 *	0.315	1.525 *	0.306	2.849 *	0.664
Single (never been married)	0.828	0.151	0.577 *	0.102	0.668	0.157
Divorced or separated	3.214 *	0.701	2.319 *	0.605	1.624	0.454
Domestic partner	0.763	0.489	1.688	0.666	2.754 *	0.918
Employment status (reference: unemployed)						
Part-time employee	0.310 *	0.109	0.203 *	0.090	0.732	0.444
Out of workforce	0.433 *	0.122	0.182 *	0.063	0.613	0.318
Full time self-employed	0.250 *	0.109	0.115 *	0.054	0.193	0.195
Employed full time for an employer	0.250 *	0.074	0.129 *	0.045	0.480	0.247
Income quintile group (reference: first quintile group)						
Second quintile group	0.610 *	0.116	0.436 *	0.083	0.553 *	0.126
Third quintile group	0.368 *	0.075	0.398 *	0.080	0.431 *	0.100
Fourth quintile group	0.312 *	0.064	0.424 *	0.083	0.442 *	0.105
Fifth quintile group	0.119 *	0.028	0.159 *	0.037	0.220 *	0.055
Constant	1.823 *	0.538	3.948 *	1.360	0.419	0.221

OR–Odds Ratio, SE–robust standard errors (with heteroscedasticity-robust asymptotic variance), * means statistical significance at 0.05.

## Appendix A

**Table A1:** Values of the Cramer’s V measure for 2014–2015 data.

Variable	Education	Gender	Marital Status	Household Composition	Status of Employment	Income Quintile	Location of Dwelling
Education							
Gender	0.0898						
Marital status	0.1768	0.1953					
Household composition	0.1039	0.1134	0.3212				
Status of employment	0.2308	0.1718	0.1755	0.1338			
Income quintile	0.2119	0.0697	0.0949	0.2269	0.1030		
Location of dwelling	0.1322	0.0337	0.1202	0.1011	0.0611	0.1115	

**Table A2:** Values of the Cramer’s V measure for 2016–2017 data.

Variable	Education	Gender	Marital Status	Household Composition	Status of Employment	Income Quintile	Location of Dwelling
Education							
Gender	0.1481						
Marital status	0.2442	0.1686					
Household composition	0.1357	0.0908	0.3615				
Status of employment	0.2951	0.1136	0.1917	0.1496			
Income quintile	0.1854	0.0341	0.0798	0.2212	0.1316		
Location of dwelling	0.1404	0.0345	0.0672	0.0870	0.1060	0.1380	

**Table A3:** Values of the Cramer’s V measure for 2018–2019 data.

Variable	Education	Gender	Marital Status	Household Composition	Status of Employment	Income Quintile	Location of Dwelling
Education							
Gender	0.1229						
Marital status	0.1894	0.1425					
Household composition	0.1353	0.0986	0.3878				
Status of employment	0.2509	0.1316	0.1960	0.1431			
Income quintile	0.1996	0.0702	0.0986	0.2443	0.1440		
Location of dwelling	0.1151	0.0394	0.0890	0.0769	0.0904	0.1481	
